# Healthcare Priorities: The “Young” and the “Old”

**DOI:** 10.1017/S0963180122000299

**Published:** 2023-04

**Authors:** Ben Davies

**Affiliations:** Uehiro Centre for Practical Ethics, University of Oxford, Oxford OX1 1PT, UK

**Keywords:** fair innings, ageism, priority-setting

## Abstract

Some philosophers and segments of the public think age is relevant to healthcare priority-setting. One argument for this is based in equity: “Old” patients have had either more of a relevant good than “young” patients or enough of that good and so have weaker claims to treatment. This article first notes that some discussions of age-based priority that focus in this way on old and young patients exhibit an ambiguity between two claims: that patients classified as old should have a low priority, and that patients classified as young should have high priority. The author next argues, drawing on a problem raised by Christine Overall, that equity cannot justify giving “old” patients low priority, since there is wide variety in the total lifetime experiences of older people, partly influenced by gender, race, class, and disability injustice. Finally, the author suggests that there might be a limited role for age-based prioritization in the context of infant and childhood death, since those who die in childhood are always and uncontroversially among the worst-off.

## Introduction

Some theorists and members of the public^1^ think age is a valid criterion for allocating health resources. A common version of this view is “microageism,” the view that if medical professionals can extend the life of either a “young” or “old” patient, they should prioritize the young patient. In addition, public health spending can target health conditions that differentially affect various age groups, or it can prioritize members of certain age groups for life-extending treatments. Although less commonly proposed, one might think that if we should prioritize young over old patients in microageism, we should also prioritize younger people at the level of public health. Call this “macroageism.”

There are two possible justifications for using age as a resource allocation criterion.^2^ The first is an efficiency justification,^3^ also called (somewhat misleadingly) “utilitarian ageism.”^4^ In this view, we should maximize the health benefit produced by a set of resources. Typically, extending the lives of old patients will produce less total good than extending the lives of young patients. In some cases, such as intensive care treatment, old patients have lower chances of benefitting from treatment. And even when they do benefit, they will generally go on to live for less additional time, and with more health complications, than young patients. Such justifications have been the subject of considerable opposition.^5^ My focus, however, is on a second justification, which appeals to equity and is sometimes called “egalitarian ageism.”^6^ In this view, we should prioritize extending younger patients’ lives—through microageism and/or macroageism—because this is fairer. From here on, then, when I talk about age-based prioritization, I assume an equity justification.

The simplest version of an argument for age-based priority assumes the following principle:

*Lifetimes Only*: Distributive justice principles apply across people’s lifetimes, and not any other times.

For the purposes of this article—to argue against standard justifications of egalitarian ageism—I assume that Lifetimes Only is true.^7^ If we care about equality, Lifetimes Only says that what we care about is equality across people’s lives (e.g., that they have equally good lives or equal opportunity across their lives). Two people’s lives can go equally well when taken as whole, even though they are never equal at any point in time, or any stage of their lives. For example, a person whose life starts off very well and then gets continuously worse may have an equally good life to someone whose life starts off very badly and gets steadily better. Lifetimes Only says there is no justice-relevant inequality here. Assuming the truth of Lifetimes Only strengthens the case for age-based priority compared with a view that also cares about shorter periods. Even if old patients have weaker lifetime claims, the possibility of time-relative claims complicates the question of overall priority. So, Lifetimes Only strengthens the case for the view that I criticize.

The structure of the article is as follows. I first outline the argument for egalitarian ageism in more detail. I then make a point that has been missed in the discussion of age-based prioritization. In comparing the young and old, we are potentially conflating two separate ideas: that “old” people take lower priority, and that “young” people take higher priority. As long as the categories of young and old are not jointly exhaustive (e.g., if there is also a group of “mature adults” or the “middle aged”), then these ideas are not equivalent.

I next turn to the main goal of the article, which is to argue that *if* we are going to adopt age-based prioritization, the first formulation—old people have lower priority—is not justified on egalitarian grounds. The second formulation—young people have higher priority—might be. The main reason for this is a divergence, which increases with age, in individuals’ welfare, opportunity and other goods that are relevant to justice. After outlining this argument and applying it to the question of giving lower priority to the old, I consider how giving greater priority to the young may avoid this problem and then briefly explore what a policy of giving priority to the young might look like. Finally, I consider two objections.

## Egalitarian Ageism

Young people have lived for less time than old people have. Thus, say egalitarian ageists, it is fair to prioritize extending their lives. Note that this does not obviously apply to interventions that are not life-extending. If a young patient will survive to old age no matter what we do, then failing to treat them may not create unfairness. From an egalitarian perspective, assuming the truth of Lifetimes Only, we will want to know how the rest of their life will turn out; it could be that even without treatment the young patient will have a better life overall than the old patient. That is not to say that a similar argument could not be developed for at least some health conditions, particularly those which are expected to have significant, negative, lifelong effects. But I will stick in my discussion to interventions without which a patient will likely die.

There are different ways we might understand the equity argument. The equity argument for age priority might appeal to:

*Equality*: Extending old people’s lives increases inequality; extending young people’s lives decreases inequality. So, we have an egalitarian reason to prefer extending young people’s lives over extending old people’s lives.^8^

*Priority*: A person who dies young is noncomparatively worse off than a person who dies in old age. So, claims to life-extending interventions are stronger for the young.

*Sufficiency*: There is some age-based threshold, which old people are above, and young people are below, which marks the point at which people have lived long enough. Those who have lived long enough have weaker claims to life-extending health interventions.^9^ The claims of those who have not had enough life are not subject to such weakening. While some understand this view as focusing purely on the amount of time someone has lived, others have thicker accounts where completeness means achieving certain distinctive kinds of good.

I return to these formulations below. In the next section, though, I show that age-based prioritization can focus on one or both of the young and old, and that these focuses are not equivalent.

## “Prioritize the Young” and “Deprioritize the Old” Are Not Equivalent

Views about age-based prioritization are sometimes arrived at by considering age groups across the lifespan.^10^ But such claims are also sometimes arrived at, or formulated, by comparing what we might think of as “young” and “old” individuals or groups. This includes empirical work on public attitudes to age-based prioritization, which asks respondents to compare patients who are distant in age. For instance, P.A. Lewis and M. Charny have respondents compare patients ages 5 and 70; 35 and 60; and 2 and 8.^11^ Respondents overwhelmingly preferred age 5 patients (94%); strongly preferred age 35 patients (80%), and slightly preferred age 8 patients (21%, with 54% unanswered). Whereas most (74%) found the first comparison easy, this dropped to 40% for the second comparison and 8% for the third. Similarly, Jana Rogge and Bernhard Kittel compare patients of ages 30 and 70^12^; Daniel Eisenberg and coauthors compare patients of ages 10 and 60.^13^ Erik Nord and coauthors suggest that support for age-based prioritization comes from public preferences for “children and young adults over elderly people,” and ask respondents to compare patients of age 20 with patients of ages 10, 60, or 80.^14^ Markku Myllykangas and coauthors simply ask respondents to evaluate the comparative claims of “children and young people” against “elderly people,”^15^ while Paul Dolan and coauthors found across several studies that “age was clearly important but there was no real consensus about when age mattered for policy and when it did not—except in the case of children versus adults.”^16^

Assume that we should use such evidence as a guide to the allocation of health resources. Comparisons of old and young patients would then give us the following claim:

*Young-Old Priority*: When deciding between “young” and “old” patients for life-extending interventions, equity requires that we prioritize young patients.

Drawing from the broadest definitions in the above-cited work, assume that “young” means “35 and under,” while “old” means “60 and older”; narrower definitions of these groups would make my point stronger. Even on these broad definitions, we are left with a third group, the “middle-aged”: those ages 36–59. Young-Old Priority does not tell us what to do when comparing young and middle-aged people, or middle-aged and old people. For both comparisons, we get two possible views. For instance, an advocate of Young-Old Priority might think we should always prioritize younger patients over older patients, whatever their ages:

*Young-Middle Priority*: When deciding between “young” and “middle-aged” patients for life-extending interventions, equity requires that we prioritize young patients.

*Middle-Old Priority*: When deciding between “middle-aged” and “old” patients for life-extending interventions, equity requires that we prioritize middle-aged patients.

Of these three claims, one might accept all of them, reject all, or accept some subset. Not all subsets are rational to accept. For instance, some might say that while we should prioritize the young over the old, we should avoid prioritizing the young over the middle-aged, or the middle-aged over the old, because priority should be given only when there is “significant” inequality. But this generates a paradox if we must choose one of a young, middle-aged, or old patient, since while the young patient should be preferred over the old patient, the young patient gets no higher priority than the middle-aged patient, who in turn gets no higher priority than the old patient. Thus, those who accept Young-Old Priority should accept at least one of Young-Middle Priority and Middle-Old Priority.

I therefore focus on two possible combinations of views, one of which is popular in the literature on age-based prioritization, and the other of which is not. The first combination is to endorse Young-Old Priority and Middle-Old Priority, but to reject Young-Middle Priority. This is essentially the Fair Innings view, and it can be summarized as:

*Deprioritize the Old*: Old patients take on a lower priority for life-extending health interventions than patients of other age groups. Age is otherwise not relevant, at least for reasons of equity.

The second combination also accepts Young-Old Priority. However, it rejects Middle-Old Priority, and endorses Young-Middle Priority, giving us:

*Prioritize the Young*: Young patients take on a higher priority for life-extending health interventions than patients of other age groups. Age is otherwise not relevant, at least for reasons of equity.

It is obvious, but important, that these ideas are different. They are also easily conflated if we only compare the claims of young and old patients. Finding public support for prioritizing a child over a 70-year-old is consistent with both views, and thus is not straightforwardly evidence for Deprioritize the Old. Similarly, philosophical approaches which aim to pump our intuitions about cases to develop ethical and policy proposals cannot solely appeal to cases that only compare young and old patients. Now, as I have acknowledged above, there is plenty of work on age prioritization that does not do this; and such work may support the conclusions about public opinion claimed by more limited comparisons. Still, a preliminary conclusion of this article is that we need to take care in using data or philosophical thought experiments which are ambiguous between whether respondents value prioritizing young patients, deprioritizing old patients, or both (e.g., through a continuous prioritarian age-weighting such as in Greg Bognar and Iwao Hirose’s discussion).^17^ If we want to test an age cutoff above which patients—classified as “old”—get reduced priority, then the best way to test this is not by comparing such patients with children or young adults, but rather with those just below the cutoff. If we want to test whether there is a general, continuous preference for younger over older patients at all life stages, then we need to make several comparisons. Finally, if the comparison made *is* between the unambiguously young and unambiguously old, then what we get to conclude about public preferences is limited to this comparison, that is, that people prioritize saving the young over the old.

From here on, I argue for a second conclusion. Even if the public prefer both Prioritize the Young *and* Deprioritize the Old, this does not mean both of those policies are justified. In the next section, I draw on work in feminist and anti-racist philosophy to argue that any view which tells us to Deprioritize the Old is not justified on egalitarian grounds. I will then suggest, however, that these arguments do not obviously undermine prioritize the Young; thus, if any version of age-based prioritization is plausible, it is not a version that looks to the end of life, but one which looks toward the beginning. I end by making some suggestions for what youth priority might look like and considering some objections.

## Deprioritize the Old Is Not Justified by Appeal to Lifetime Equity

Christine Overall has considered the idea of human enhancements targeting the aging process, and thus potentially offering radical extensions to the human lifespan.^18^ Drawing on earlier work in feminist philosophy,^19^ and echoing other discussions,^20^ Overall responds to the charge that such radical lifespan extension would be unjust because it favors those who are already old. She notes that this critique makes the error of assuming “that it can only be young people who have not yet had the opportunity to enjoy the good of human life.”^21^ The reason this is an error is, in large part, because of various forms of injustice, including but not limited to racism, sexism, and disability discrimination. Similarly, Ian Dey and Neil Fraser^22^ ask why we should look solely to age as a marker of those who are better off in lifetime terms, and not to other demographic facts such as gender, class, or disability status. More recently, Luis Cordeiro-Rodrigues and Cornelius Ewuoso^23^ have suggested that using age in triage protocols is a form of “racism without racists,” since such approaches systematically disadvantage black patients—although their argument is framed as engaging with consequentialist views, they also discuss arguments that would fall under “egalitarian” ageism.

My interest is not so much in Overall’s conclusions about radical life extension, but rather in the general form of the argument she makes against this justice-based critique of extending the lives of the old. It is an argument that can be generalized to think about age-related prioritization more generally.

Earlier in the article, I outlined several different interpretations of an equity justification for age-based prioritization, based respectively on appeals to equality, priority, and sufficiency. Each interpretation began with an empirical claim. For instance, the priority version claims that those who die young are worse off than those who die old. The sufficiency version posits the existence of a threshold, in which old people are above and young people (currently) below, that marks the point at which one has “lived long enough.” The equality version claims that extending the lives of old people increases inequality, whereas extending the lives of those who would otherwise die young decreases it.

The central problem, following Overall and others, is that these empirical claims are all false, at least on many ways of understanding the terms “young” and “old.” They are false because for most *adults* (I come to children in a moment), for any two ages we choose, there will be many people in the younger age group who would have had better total lifetimes if they died today than many people in the older age group. For instance, if I died today at the age of 34, I would not have had a worse lifetime overall than some people aged 70 or older. Thus, if a health system had to choose between extending my life and some randomly chosen 70-year-old, we cannot know whether saving the 70-year-old would increase inequality, and it might not be true that the younger patient (me) would be noncomparatively worse off in lifetime terms than the older patient.

These observations hold almost whatever currency of justice we choose. There are 34-year-olds who have had more total lifetime welfare than some much older people. They have had more opportunities already, and they have enjoyed the use of a greater total amount of resources. On some obvious measures of health, such as Quality-Adjusted Life Years (QALYs), a young person who has had very good health may have enjoyed a greater total number of QALYs than a much older person with considerably worse health.

The falsity of the sufficiency interpretation is different in structure. The sufficiency view claims that there is some age at which people have had a complete life, or lived long enough. Consider first a sufficiency view that sets its age threshold at a point where individuals have had a good enough lifetime total of some currency of justice, such as welfare. Say that the chosen threshold is 70. The sufficientarian who wants to endorse Deprioritize the Old faces a dilemma: is 70 the relevant threshold because of the cumulative well-being of the best-off 70-year-olds, of the average, or of the worst-off? If it is the best-off or the average, then there will be some, perhaps many, 70-year-olds who have not yet reached the cumulative total that is supposed to ground a complete life. If it is the worst-off, then the best-off, and many others, will have reached that threshold long before 70. Aside from the apparent arbitrariness of choosing any particular age as the relevant threshold, no age marks a threshold where all, or even most, people actually reach a particular level of well-being, opportunity, or resource use.

One potential currency of justice where old people invariably fare better than young people is age itself: if the mere number of years lived were a currency of justice, then it is true by definition that old people are better off than those who die young. But age itself is either not a currency of justice, or it is one whose importance for justice is dwarfed by other considerations. The mere fact that someone has lived a long time is not all that significant to their claims.

A second potential currency,^24^ which relates most obviously to certain kinds of “narrative” view about a complete life (which I have classed above as sufficientarian), concerns the idea that there are certain central human goods that can only be achieved at certain ages. Proponents of such a view have two options. One option is to suggest certain age-specific goods that are common to all individuals.^25^ That might include, for instance, seeing one’s grandchildren grow up, or enjoying some years of retirement after a fulfilling career. However, these goods can come at significantly different ages, including ages at which one is not obviously “old.” Moreover, some people do not want these goods at all: if “watching one’s grandchildren grow old” is a *necessary* component of a complete life, then the person who decides not to have children will never have a complete life. Of course, we might fix this issue by saying that one must have had the opportunity for such goods. But this is not obviously much better. Not everyone *has* had these opportunities upon reaching old age; and this shift does not solve the problem of those who fulfill these experiences much younger or much older than is typical.

An alternative option is to suggest a wide variety of *possible* components of a complete life and say that the relevant components depend on the individual. At an extreme, we might say that a person is “old,” and thus that their claims to life-extending care have lesser weight, when they have lived a complete life by their own lights.^26^ But this is unhelpful for health policy: providers of life-extending care cannot go round asking people whether they have lived a complete life by their own lights; and such an approach would completely rule out age as a criterion for macroallocation: there is no particular age where we can be sure that all or even most people will regard their life as complete. Thus, the fact that someone is old is neither intrinsically relevant to justice, nor a good proxy for facts, which are intrinsically relevant.

The reasons for the disparities we see within age groups, and between the well-off young and badly-off old, are various. Some old people have had lives of poor quality due to injustice, as Overall notes. Such injustices may be personal; often they are structural. Perhaps the empirical claims about old and young people made in by the egalitarian, prioritarian, and sufficientarian versions of the equity argument would be more plausible if we controlled for various demographic factors, such as economic class, race, gender and disability status. But they are not true within any society, and certainly not at a global level, if we ignore those factors.

However, others may have had bad lives in part due to their own choices. Greg Bognar (personal communication) suggests that my argument may ignore this important fact. Perhaps so long as everyone has a reasonable *chance* of living a good life, then the fact that some old people have had worse lives than some young people does not matter.^27^ If people end up badly off through their own choices, maybe this is not unjust. This is a sufficientarian view with opportunity as its currency.

There are two points to make about this suggestion. First, it is not obvious that responsibility is relevant to health justice to such a comprehensive degree. Second, even if the normative claim is true, the antecedent of the conditional is false. It is *not true* that everyone has had a reasonable chance of living a good life, and that we can typically explain failure to do so by individual choice.

This raises an important point about my argument. I am not claiming—at least, not here—that it is necessarily unjust to use old age as a criterion in healthcare allocation. By necessarily unjust, I mean that it would be unjust in all societies, in all realistic situations. My claim is more limited, though still fairly strong: I claim that in many contemporary societies, including my own society of the UK, such a policy is unjust because of the actual injustice that many older people have faced. My interest in this article is in actual policies that might be adopted. Thus, I conclude this section by reiterating that the policy “Deprioritize the Old” is unjust, or at least not justified by reference to lifetime equity. This argument also raises problems for views such as Norman Daniels’s Prudential Lifespan Account, which asks us to see age-based interpersonal choices in the light of what we would choose *intra*personally across a lifespan.^28^ Where people have very different qualities of life, opportunity, and so on, there are considerable problems in translating intrapersonal prudence to interpersonal fairness.^29^

Amassing a significant number of life-years may be “supremely valuable,”^30^ but its value is primarily, perhaps solely, instrumental. Since different individuals have significantly different opportunities and outcomes in converting life years into other goods, I suggest that Deprioritize the Old cannot be justified by reference to lifetime equity.

## Prioritize the Young *Might* Be Just

The objection to Deprioritize the Old which is at the heart of my argument in the previous section relies on an empirical claim of increasing divergence. In terms of total lifetime well-being, opportunity, and resources enjoyed, the gap between the best- and worst-off in a particular generation grows as people age; thus, age is not the only, nor the most, relevant fact about people.^31^ Take QALYs as an example. Imagine two people. One experiences good health, leaving them with an average of 0.95 QALYs per year. The other experiences significant, but by no means the worst possible, health burdens for much of their life—for simplicity, from birth—leaving them with an average of 0.6 QALYs per year. After they have lived for 1 year, there is a disparity of .35 QALYs: the equivalent of around a third of a year of full health. At age 90, the disparity is 31.5 QALYs: the better-off person has enjoyed 85.5 QALYs across their life, while the worse-off person has had only 54. Indeed, in lifetime QALY terms, the better-off individual already had a greater cumulative total at the age of 57 than the other individual will have across their whole life. Although less formally measurable, the same is true of people who have different average levels of well-being more generally, or who enjoy different levels of resources. Opportunity is perhaps less straightforwardly cumulative in nature: but it is often true that those who enjoy opportunities early on in life get access to further opportunities, while those who faced disadvantages do not.

As well as this cumulative divergence in lives, many societies are marked by significant inequality even from birth. From a lifetime perspective, an infant from a privileged background who lives to old age is much more likely to be among the best off in lifetime terms than an infant from a disadvantaged background. Now, though, focus on a subset of individuals: those who face the possibility of death in their infancy or childhood. From a cumulative lifetime perspective, someone who dies in infancy is among the worst-off even if, had they survived and lived to old age, they would have been among the best off. If our better-off individuals above had died at age 2, they would have accumulated only 1.9 QALYs across their whole lifetime, far less than the individual who enjoys fewer QALYs per year, but lives far longer.

The person who dies very young is, in lifetime terms, very badly off in a way that is relevant to prioritarians, and worse off than most others in the sense that egalitarians care about. On any plausible view of lifetime sufficiency, those who die in infancy have not crossed the relevant threshold. The various value disagreements that trouble axiologically substantive complete life views, the potential arbitrariness that attaches to choosing a particular age as the threshold: these do not trouble the claim that those who die very young have not had a sufficiently long life. So, whereas the issue of divergence creates a significant problem for an age-based priority view that focuses on the old, it does not obviously do so for a view focusing on the young.

It is worth briefly noting the distinction between Prioritize the Young and an alternative view, proposed by Persad et al., the “complete lives system.”^32^ The complete lives system prioritizes allowing people to live complete lives. As such, a key difference between it and Prioritize the Young is that it gives priority to adolescents and young adults over infants, in part because the latter have “invested” in their lives in a way that gives achieving a complete life greater value. Persad et al claim that the aim of their system is to “achieve complete lives.” A superficial understanding of this is to see it as a qualified version of a “headcount” sufficientarian approach, where what matters is getting people over the threshold of a complete life. It is therefore worth noting a point raised by Hirose and Bognar, that such a view gives only tentative support to prioritizing younger patients (even adolescents and young adults). For instance, assuming that a complete life is set at 70, a view which genuinely prioritized complete lives would prefer to extend a 69-year-old’s life by 1 year than to extend a 20-year-old’s life by 49 years. This diverges from both efficiency and equity-based reasons for age-related priority.^33^ I suspect that advocates of the complete lives view would not in fact endorse this conclusion,^34^ and that it is really a form of egalitarian ageism with more fine-grained categories than those I consider.

Thus, I make the following conditional claim. *If* age-based prioritization is justifiable, it is justifiable only in the form of Prioritize the Young, not Deprioritize the Old. This is consistent with the view that age-based prioritization is not justifiable at all.

## The Content of “Youth Priority”

A policy of Prioritize the Young needs to stipulate at least two things: who counts as young, and in what respects they should be prioritized over patients who are not young. I have suggested that the most plausible role for age in health prioritization is as an easily judged variable that has no intrinsic moral value, but which imperfectly tracks facts that do have intrinsic moral value. At very young ages, there is a further pragmatic convergence: on almost any view of lifetime fairness, those who die in infancy or early childhood had a very strong claim to have their lives extended. As we get older, this pragmatic overlap weakens. A very well-off 20-year-old may have already reached a point where they are no longer the victim of a serious lifetime *well-being* inequality if they were to die, but will likely have failed to reach lifetime sufficiency on a view where a complete life requires certain age-specific goods or opportunities. Additionally, at 20, people’s lives have had quite some time to nonculpably diverge in quality, so that without a significant investigation into the nature of someone’s life so far (an investigation that is pragmatically beyond any health system) we cannot be sure that the patient in front of us has crossed a relevant threshold.

Thus, as we look at increasingly older “young” patients, we face two kinds of uncertainty: normative uncertainty about what the correct view of fairness is and empirical uncertainty about the status of a particular patient or patient group within that view of fairness. This speaks in favor of adopting a fairly low threshold for classifying patients as “young.” In doing so, we must recognize that some who are thereby classified as “not young” would have qualified for prioritization if we had more information. Those who fall on the wrong side of the prioritization threshold in this case are treated like the rest of the adult population; this differs from those who are miscategorized in Deprioritize the Old. Still, I grant that this is an ethical strike against what seems to be the most plausible version of Prioritize the Young.

Assume, then, that we can arrive at a suitable threshold for determining who counts as “young.” What does Prioritize the Young imply in practice? I suggested at the start of the article that there are at least two levels at which such a principle may operate: the microlevel and the macrolevel. At the macrolevel, Prioritize the Young would suggest giving what would otherwise be disproportionate funding to conditions that predominantly threaten the lives of the young (even prior to considering the relative effectiveness of such treatments) and disproportionately targeting funding for life-saving interventions that can benefit all patients to young populations.

What about the microlevel? Much empirical work on attitudes toward age considers scenarios where we can save only one of two patients who differ in age only. Such scenarios are not all that realistic as representative of real-life decision making. Still, Prioritize the Young would suggest that we should give additional weight to the fact that someone is young, but otherwise be indifferent between saving the lives of patients who differ in age only. Perhaps more realistic would be to consider age as one factor among many in cases of triage—such as admission to ICU during emergency situations where more patients can benefit from intensive care than can be admitted—or on waiting lists.^35^

## Objections

I finish this article by considering two objections.

### The Scope of Justification

The first objection concerns the structure of my response to various ways of understanding Deprioritize the Old. I suggested, for instance, that a view that stipulates certain goods as components of a complete life is not plausible because not everyone who reaches old age will have achieved, or had opportunity to achieve, those goods. However, one might object that if my interest is in real-world health systems, it is unreasonable to insist on a standard that is 100% accurate. No way of prioritizing health interventions could meet this criterion. What we need, one might say, is to build our health care prioritization around reasonably reliable indicators.

According to this objection, although not all old people have lived complete lives (or have met some alternative criterion of justice), the proportion who have done so is large enough that a health system, which categorizes *all* old adults as having lived complete lives will be more just than one which does not. Some individuals who have not yet lived a complete life will be unjustly treated as though they have. But it is, so this objection says, inevitable that any allocation system will make regrettable errors. The aim is to minimize them.

This objection is not equally plausible as a defense of microageism and macroageism. It is more plausible that macrolevel decisions, such as those taken by governments or large public health bodies, cannot take account of every morally relevant feature of each individual^36^; rather, those making such macrolevel decisions need to come up with rules that are better than plausible alternatives. This is not to say that the objection has *no* force at the microlevel. Even microlevel decisions cannot take every detail into account; it might still be good for those operating in emergency triage, for example, to have generic rules that they can apply easily, and which will tend to produce fairer results than alternatives. Still, other kinds of microlevel decisions seem more amenable to consideration of other facts, and to recognize that although an individual fits into a particular category (such as “old”), they do not fit the normatively typical stereotype of that category. Thus, even if the objection works, it has some limits on its scope.

However, the objection faces a broader issue. While it is true that no method of allocating health resources can expect to avoid moral error, it is of greater importance to avoid what John Harris has influentially termed “double jeopardy,” or the compounding of injustice.^37^ In other words, those who lose out on a policy like “Deprioritize the Old” include those who have *already* borne the brunt of social injustice across their lives. While it is impossible to avoid all miscategorization, it is of particular importance to avoid miscategorization that compounds injustice. Thus, I suggest that even though no allocation principle can achieve perfect justice, Deprioritize the Old is likely to make things overall worse, not better.

## The Most Disadvantaged Do Not Make It

Govind Persad and Steven Joffe^38^ defend age-based prioritization against the kind of argument I have made by noting that similar injustices also affect how long people live. Their argument is focused on access to ventilators during the COVID-19 pandemic, but might be generalized.^39^ For instance, they note that “78% of US COVID-19 decedents younger than 21 were minorities,” and that “Disparities in COVID-19 deaths in the UK similarly appear to intensify at younger ages.” They explain that this is because the most disadvantaged are unlikely to have survived to old age. Thus, we should endorse both Prioritize the Young *and* Deprioritize the Old, because failure to do so will result in more premature deaths, which will disproportionately affect those who have been unjustly disadvantaged.

It is worth noting that Persad and Joffe are responding to an argument from Dave Archard, who is explicitly arguing for the claim that it is “wrong to prioritize younger patients with COVID-19.”^40^ The argument I have given offers some limited support for the Persad-Joffe position, since it suggests that it may be acceptable to prioritize *young* patients. That age-based priority should aim, as Persad and Joffe suggest, to mitigate “life-shortening disadvantage” is consistent with a focus on the young rather than on the old, or with a lifelong prioritization of younger over older patients.

Archard suggests that some deserve to live more than others due to their choices, suggesting that “it matters what kind of life has been led and might still be led.” I think Persad and Joffe are right to reject such an appeal to personal responsibility in emergency situations such as ventilator allocation. But it is worth noting that Archard’s phrasing here might be interpreted differently: although Persad and Joffe are right to say that one serious effect of unjust disadvantage is its “life-shortening” effect, we should also be concerned with life-*worsening* disadvantage, which affects even those who make it to old age. This is the sense in which it “matters what kind of life has been led.” Even if those who make it to old age are not the very worst-off (since the very worst-off disproportionately die in their youth) it is possible to live to be elderly and be very badly off.^41^ How serious this is will depend in part on what needs an age-based prioritization society refuses to treat; but premature death is not the only injustice worth considering.

## Conclusion

Age-based prioritization—which in practice is often interpreted as giving lower priority to patients and patient groups classified as old—often draws on considerations of lifetime fairness. I have aimed to show, first, that it is important to distinguish between different ways of classifying a population by age, and not to assume that all forms of age-based prioritization are equivalent. Second, I have argued that if we are to give a role to age in allocating health-related resources, that role should be focused on giving greater priority to young patients, rather than lower priority to old patients. This second argument is importantly conditional; it may be that, all things considered, it is better to avoid fairness-based arguments for age-based priority altogether.

## NOTES

1.

Harris J. The Value of Life: An Introduction to Medical Ethics. London: Routledge; 1985:94 ; Daniels N. *Am I My Parents’ Keeper?* An Essay on Justice Between the Young and the Old. Oxford: OUP; 1988 ; Veatch RM . Justice and the economics of terminal illness. The Hastings Centre Report 1988;18:34–40 3146557; Kappel K , Sandøe P. QALYs, age and fairness. Bioethics 1992;6:297–316 1165209210.1111/j.1467-8519.1992.tb00208.x; Kappel K , Sandøe P. Saving the young before the old: A reply to John Harris. Bioethics 1994;8:84–92 1165737710.1111/j.1467-8519.1994.tb00243.x; Callahan D. Setting Limits: Medical Goals in an Ageing Society. Washington, DC: Georgetown University Press; 1995 ; Nilstun T , Ohlsson R. Should health care be rationed by age? Scandinavian Journal of Social Medicine 1995;23:81–4767622210.1177/140349489502300201; Singer P , McKie J , Kuhse H , Richardson J. Double jeopardy and the use of QALYs in healthcare allocation. Journal of Medical Ethics 1995;21:144–50767427810.1136/jme.21.3.144PMC1376689; Nord E , Street A , Richardson J , Kuhse H , Singer P. The significance of age and duration of effect in social evaluation of health care. Health Care Analysis 1996;4:103–111016276110.1007/BF02251210; Persad G , Wertheimer A , Emanuel EJ . Principles for allocation of scarce medical interventions. The Lancet 2009;373:423–3110.1016/S0140-6736(09)60137-919186274; Fleck LM . Just caring: In defense of limited age-based healthcare rationing. Cambridge Quarterly of Healthcare Ethics 2010;19:27–37 2002580010.1017/S0963180109990223; Lazenby H. Is age special? Justice, complete lives and the prudential lifespan account. Journal of Applied Philosophy 2011;28:327–40; Tallman R. Valuing lives and allocating resources: A defense of the modified youngest first principle of scarce resource distribution. Bioethics 2014;28:207–132284566810.1111/j.1467-8519.2012.01994.x; Gipe K , Kelstein S. Let us be fair to 5-year-olds: Priority for the young in the allocation of scarce health resources. Public Health Ethics 2018;11:325–35; Persad G , Joffe S. Allocating scarce life-saving resources: The proper role of age. Journal of Medical Ethics 2021;47:836–810.1136/medethics-2020-10679233753473; See Bognar G. Fair innings. Bioethics 2015;29:251–612490944910.1111/bioe.12101 for discussion of empirical work on public attitudes; See also Battin MP . Age rationing and the just distribution of health care: Is there a duty to die? Ethics 1987;97:317–401165887810.1086/292841; Hardwig J. Is there a duty to die? Hastings Center Report 1997;27:34–42 9131351; and Though see Kuylen MNI , Kim SY , Keene AR , Owen GS . Should age matter in COVID-19 triage? A deliberative study. Journal of Medical Ethics 2021;47:291–53368791710.1136/medethics-2020-107071PMC7944418.

2.

Bognar G. Age-weighting. Economics and Philosophy 2008;24:167–89.

3.

Tschuyia A. QALYs and ageism: Philosophical theories and age weighting. Health Economics 2000;9:57–68 1069476010.1002/(sici)1099-1050(200001)9:1<57::aid-hec484>3.0.co;2-n.

4. See note 1, Nord et al. 1996.

5.

Leach Scully J. Disability, disablism, and COVID-19 pandemic triage. Journal of Bioethical Inquiry 2020;17:601–53284083210.1007/s11673-020-10005-yPMC7445721.

6. See note 1, Nord et al. 1996 and note 3, Tschuyia 2000.

7. In fact, I think Lifetimes Only is false. We do have justice-based reasons to care how well or badly off people are at particular times, in a way that is not reducible to the effect of those times on their life overall. See McKerlie D. Equality and time. *Ethics* 1989;99:475–91; Jecker N. Age related inequalities in health and healthcare: The life stages approach. Developing World Bioethics 2018;18:144–552850842210.1111/dewb.12148; and Davies B. Health(care) and the temporal subject. The Ethics Forum 2018;13:38–64 .

8. The equality justification of age-based priority also tells us, taken by itself, that extending older people’s lives is in one way bad even when they do not compete with younger people. This is a form of “leveling down,” and can be avoided in the typical way, by adopting a pluralist view where equality is not the only thing that matters.

9. Like the egalitarian view, the stronger version of this view—that those who have had enough life have no claim whatsoever to more life—implies that we have no reasons of justice to extend an old person’s life. Unlike the egalitarian view, it is not so clear that this can be solved pluralistically. While one might, of course, hold that we have reasons of beneficence to benefit people, it is not clear whether a publicly funded health system can be justified on grounds of beneficence rather than justice. Old people at least have a more secure claim to life-extending interventions, I suggest, if their claims are grounded in justice.

10. See note 1, Nord et al. 1996. Also Jelsma J , Shumba D , Hansen K , de Weerdt W , De Cock P. Preferences of urban Zimbabweans for health and life lived at different ages. Bulletin of the World Health Organisation 2002;80:204–9PMC256775411984606; Olsen JA . Priority preferences: “End of life” does not matter, but *total* life does. Value in Health 2013;16:1063–62404135610.1016/j.jval.2013.06.002; Petrou S , Kandala N , Robinson A , Baker R. A person trade-off study to estimate age-related weights for health gains in economic evaluation. Pharmacoeconomics 2013;31:893–907 2399038910.1007/s40273-013-0085-y; Neuberger J , Adams D , MacMaster P , Maidment A , Speed M. Assessing priorities for allocation of donor liver grafts: Survey of public and clinicians. BMJ 1998;317:172–5966589510.1136/bmj.317.7152.172PMC28607 offers an age-based principle that focuses on prioritizing the young rather than deprioritizing the old, asking respondents whether “patients aged under 5 years should be given priority” in liver transplantation.

11.

Lewis PA , Charny M. Which of two individuals do you treat when only their ages are different and you can’t treat both? Journal of Medical Ethics 1989;15:28–32 292678410.1136/jme.15.1.28PMC1375759.

12.

Rogge J , Kittel B. Who shall not be treated: Public attitudes on setting health care priorities by person-based criteria in 28 nations. PLoS One 2016;11:1–15 10.1371/journal.pone.0157018PMC490056327280775.

13.

Eisenberg D , Freed, GL , Davis MM , Singer D , Prosser LA . Valuing health at different ages. Applied Health Economics and Health Policy 2011;9:149–562150662110.2165/11587340-000000000-00000.

14. See note 1, Nord et al. 1996.

15.

Myllykangas M , Ryynänen O , Kinnunen J , Takala J. Comparison of doctors’, nurses’, politicians’ and public attitudes to health care priorities. Journal of Health Services Research & Policy 1996;1:212–61018087310.1177/135581969600100406.

16. Dolan P, Edlin R, Tsuchiya A. *The relative societal value of health gains to different beneficiaries.* HEDS Discussion Paper 08/12. White Rose Research Online; 2008; available at http://eprints.whiterose.ac.uk/10902/ (last accessed 23 May 2022).

17. Bognar G, Hirose I. *The Ethics of Health Care Rationing: An Introduction.* Abdingdon: Routledge; 2014:94.

18.

Overall C. Life enhancement technologies: Significance of social category membership In: Bostrom N , Savulescu J , eds. Human Enhancement. Oxford: OUP; 2009:327–40; Overall C. Aging , Death and Human Longevity. London: University of California Press; 2005 .

19. Bell NK. If age becomes a standard for rationing health care. In: Holmes HB , Purdy LM , eds. Feminist Perspectives in Medical Ethics. Indiana: Indiana University Press; 1992:82–90 ; Silvers A. Aging fairly: Feminist and disability perspectives on intergenerational justice. In: Walker MU , ed. Mother Time: Women, Aging, and Ethics. Lanham, MD: Rowman & Littlefield; 1999:203–26; Nelson JL , Nelson HL . Justice in the allocation of healthcare resources: A feminist account. In: Susan W , ed. Feminism and Bioethics: Beyond Reproduction. New York: OUP; 1996:351–70; Nelson JL. Death’s gender. In: Walker MU, ed. *Mother Time: Women, Aging, and Ethics.* Lanham, MD: Rowman & Littlefield; 1999:113–29; See also Nielsen L. Contractualist age rationing under outbreak circumstances. *Bioethics* 2021;35:229–36. An argument similar to mine seems to be mentioned by Lesser H. *Ageing and Autonomy.* Aldershot: Ashgate; 1998, cited in Rivlin MM. Why the fair innings argument is not persuasive. *BMC Medical Ethics* 2000;1:1–6.

20.

Clark Miller S. The invisibility of gender: A feminist commentary on age-based healthcare rationing. Journal of Philosophical Research 2005;30:263–74.

21. See note 18, Overall 2009, at 335.

22.

Dey I , Fraser N. Age-based rationing in the allocation of health care. Journal of Aging and Health 2000;12:511–37, at 5191150373010.1177/089826430001200404.

23. Cordeiro-Rodrigues L, Ewuoso C. Racism without racists and consequentialist life-maximizing approaches to triaging. *Bioethics* 2022;36:243–51.

24. There are other options, such as the view outlined by Williams A. Intergenerational equity: An exploration of the ‘fair innings’ argument. *Health Economics* 1997;6:117–32, that fair innings should reference not chronological age, but “quality-adjusted life expectancy” (QALE), that is, the number of QALYs one can expect to enjoy across one’s life. Whatever the merits of such a view, my interest in this paper is on the use of age either as the direct currency of justice or as a suitable proxy, and Williams explicitly acknowledges that chronological age is not a good proxy for QALE.

25. See note 1, Callahan 1995.

26.

Rivlin MM . Can age-based rationing of health care be morally justified? Mount Sinai Journal of Medicine 1997;64:113–99057468, cited in Dey, Fraser 2000, at 519 (see note 22); Jecker N. Should we ration health care? Journal of Medical Humanities 1989;10:77–90 1029699410.1007/BF01137571, at 84. Callahan D. What Kind of Life: The Limits of Medical Progress. New York: Simon and Schuster; 1990:153 , cited in Bell 1992 (see note 19). See note 1, Tallman 2014.

27. Caplan A. Is it wrong to prioritise younger patients with COVID-19? No. *BMJ* 2020;369:2.

28. See note 1, Daniels 1988; Persad, Joffe 2021.

29. See note 1, Lazenby 2011. Also Davies B. Ageing and terminal illness: Problems for Rawlsian justice. Journal of Applied Philosophy 2017;35:775–89.

30. See note 1, Persad et al. 2009.

31. See note 19, Nelson 1999.

32. See note 1, Persad et al. 2009, at 428. See also Emanuel EJ , Persad G , Upshur R , Thome B , Parker M , Glickman A , et al. Fair allocation of scarce medical resources in the time of COVID-19. The New England Journal of Medicine 2020;382:2049–553220272210.1056/NEJMsb2005114; note 1, Tallman 2014.

33. See note 17, Bognar, Hirose 2014.

34. See note 1, Gipe, Kerstein 2018, at 328.

35. A further question, beyond the scope of this article, is what a policy such as Prioritize the Young means for young patients whose lives can be extended, but not by much, for example, young patients with incurable terminal illnesses. One possibility is that the rationale behind Prioritize the Young also speaks in favor of what would otherwise be disproportionate spending on even small extensions to the lives of such patients, or to small improvements in quality, because they are unavoidably among the lifetime worst-off.

36. Wolff J, de Shalit A. *Disadvantage.* Oxford: OUP; 2007.

37.

Harris J. QALYfying the value of life. Journal of Medical Ethics 1987;13:117–23366903610.1136/jme.13.3.117PMC1375658.

38. See note 1, Persad, Joffe 2021.

39. Though see note 19, Nielsen 2021.

40. Archard D. Is it wrong to prioritise younger patients with COVID-19? Yes. *BMJ* 2020;369:1–2.

41. See note 20, Clark Miller 2005. See also note 19, Nelson, Nelson 1996, at 361–2. Also Jecker N. Age-based rationing and women. *Journal of the American Medical Association* 1991;266:3012–5.

